# Revealing the Pb Whisker Growth Mechanism from Al-Alloy Surface and Morphological Dependency on Material Stress and Growth Environment

**DOI:** 10.3390/ma15072574

**Published:** 2022-03-31

**Authors:** Matic Jovičević-Klug, Tim Verbovšek, Patricia Jovičević-Klug, Barbara Šetina Batič, Bojan Ambrožič, Goran Dražić, Bojan Podgornik

**Affiliations:** 1 Max-Planck-Institute für Eisenforschung GmbH, Max-Planck-Straße 1, 40237 Düsseldorf, Germany; 2 Institute of Metals and Technology, Lepi Pot 11, 1000 Ljubljana, Slovenia; tim.verbovsek@imt.si (T.V.); barbara.setina@imt.si (B.Š.B.); bojan.podgornik@imt.si (B.P.); 3 Jožef Stefan International Postgraduate School, Jamova Cesta 39, 1000 Ljubljana, Slovenia; goran.drazic@ki.si; 4 Center of Excellence in Nanoscience and Nanotechnology, Jamova Cesta 39, 1000 Ljubljana, Slovenia; bojan.ambrozic@ijs.si; 5 National Institute of Chemistry, Hajdrihova Ulica 19, 1000 Ljubljana, Slovenia

**Keywords:** aluminum alloy, metallic whiskers, residual stress, crystal growth, oxidation

## Abstract

Spontaneous metallic Pb whisker formation from Pb and Bi containing Al-alloy’s surfaces is a newly discovered phenomenon. The whiskers display unique formations, growth and morphology, which give the opportunity to be applied for specialized sensor and electronics applications. Within this work, the impact of environmental conditions (gas composition and moisture) is investigated and correlated with the modification of whisker evolution and growth dynamics. Furthermore, the residual stress state of the aluminum matrix using deep cryogenic treatment is modified and used to further increase whisker nucleation and growth by up to three- and seven-fold, respectively, supported by quantitative results. The results of this paper indicate the possibility to manipulate the whisker not only in terms of their kinetics but also their morphology (optimal conditions are 20% O_2_ and 35% humidity). Such features allow the tailoring of the whisker structure and surface to volume ratio, which can be optimized for different applications. Finally, this research provides new insight into the growth dynamics of the whiskers through in situ and ex situ measurements, providing further evidence of the complex nucleation and growth mechanisms that dictate the spontaneous growth of Pb whiskers from Al-alloy 6026 surfaces with growth velocities up to 1.15 µm/s.

## 1. Introduction

The spontaneous growth of metallic whiskers is a long-lasting topic that has and continues to baffle scientists to this day [1,2,3]. As the dominating “plague” in electronics, whiskers were one of the main reasons for device failures by creating shortcuts between isolated elements of electronic circuits [4]. Research on whiskers dates as far back as the 1950s, when National Aeronautics and Space Administration (NASA) first concluded that these miniature single-crystal rods (whiskers) formed on Sn-platted connectors were the main reason for device failures [5]. Further research from NASA also proved that whisker formation is not limited to Sn but can be found in a variety of metals (Al, Ag, Au, Bi, In, Pb and Zn) and alloys with a predisposition to those with low melting temperatures [6]. With consistent experimental research, the basic principles of whisker formation were resolved, which allowed the successful mitigation of these in Sn-based alloys through the addition of Pb. However, with European legislation, Restrictions on Hazardous Substances (RoHS) [7], the use of such mitigation processes was prohibited, thus reopening research into the whisker growth phenomenon. To no surprise, whisker formation has become a considerable challenge for many industries, as they have the potential to cause the failure of components leading to events such as satellite failure [5], pacemaker [8] and other medical device failures [9], nuclear plant shutdowns [10], car defects [11,12] and increased defect rates of various electrical components [4,13,14] as well as batteries [15]. For this reason, many other mitigation strategies are developed, with the mainstream using conformal coatings, which are considered to work well for most components. However, many researchers still indicated that whiskers can penetrate the coating over time or when the coating degrades or is not properly covering the Sn material [5]. The re-emerging interest in understanding whisker formation is further emphasized by the latest report from PERM (Pb-free Electronics Risk Management), indicating the large occurrence of whiskering (up to 40%) in assembly lines of aerospace, defense and harsh environment organizations [16].

Despite the extensive past research on metallic whisker formation, there is a considerable inconsistency in the understanding of whiskers growth. Several theories are proposed to explain the daunting whisker nucleation and growth for several metallic systems [1,2,3], with a majority following the explanation of stress-induced growth [3,17,18]. The stress-induced theory is supported by many researchers that have proven that stress originating from material deposition [19,20], the formation of intermetallic phases [21,22,23], oxidation [24,25,26] or externally applied stress [27,28,29,30] causes whisker growth. However, whisker formation is not homogeneous nor simultaneous. Whiskers tend to grow from the same material from minutes to months or even years [31], making a prediction of their growth through various models elusive. Furthermore, the varying incubation time for whiskers at different positions from the same samples have erupted many contradictions to established theories and proposals for additional theories that intend to explain stochastic growth [8,32,33,34,35,36,37]. Nevertheless, most of the research has provided significant input regarding growth mechanisms but limited insight into the driving forces and energies that govern whisker formation. Most studies describe the countermotion of atoms and vacancies to be the mechanism at play [1,2,3]. However, in many cases, the motion explains only the volume transmission and not the underlying driving force that dictates the directionality of the atomic motion nor the morphologic preference of elongated rods, as spheres and non-straight forms should generally be energetically preferable. Researchers proposed that the oxides at the sides of the opening or preferentially growing crystals confine the growth direction, thus leading to the typical whisker shapes [24,38]. However, many researchers provided experiments that contradict such reasons for whisker formation [1,2,3].

In our investigated aluminum alloy system (EN AW 6026), we discovered that Pb whiskers grow from specific Bi-Mg-Pb solid pools in complicated forms and display unique growth behaviors [39]. Further details about the microstructural characteristics of the investigated material are provided in [40]. The driving force for whisker growth was determined to originate from the generated stress by the oxidation of surrounding Bi phases. The generated stress is imposed on the separated pure Pb regions that are compressed, leading to the formation of whiskers. One of the main surprises of our previous work is the funnel growth that indicates that the opening of the growing root is smaller than the final whisker width, in some cases even more than 10-fold, making the confined oxide explanation rather weak. Furthermore, many whiskers displayed unique prior hillock growth in very large and strongly faceted structures, which provides a counterpoint to the simplistic models of perpendicular atom diffusion to the stressed material from below as well as the spherical growth caused by equal atom diffusion. Finally, the structures displayed varying growth modes within the same whiskers, which could be interchangeable and provide no direct correlation with the final or beginning growth stages of the whiskers. In our prior research, we explained the growth through the formation of nanocrystallites during the processing of the material that later fuses and reorients them through oriented attachment during the material motion caused by localized internal stresses. The transition from a dynamic atomistic to nano building-blocks model allows the tools to explain the different morphologies and structures that are predefined by local stress and the available material from the pool reservoirs. For more details, the reader is encouraged to read the previous work [39].

In this work, the nucleation and growth behavior of whiskers from aluminum alloy is explored regarding the dependency of the incubation environment in order to understand the underlying mechanism for the formation of whiskers. For these purposes, variation in oxygen content and humidity are performed as these two main components are considered to be the dominant reasons for interaction with the surface of the material. The selected environments range from an excessively high oxidative environment and a high humid environment, to a simulated extraterrestrial environment (such as the Mars atmosphere) in order to also explore whisker stability and interaction with the storage environment. Additionally, the internal stress of the holding aluminum matrix is explored and could additionally contribute to the increased whisker growth and varying incubation time of individual whiskers. The results are statistically evaluated and correlated to understand the dependency on selected environmental factors, which could prevent or enhance whisker nucleation and growth. Furthermore, this research explores growth dynamics and restructuring under different conditions using scanning and transmission electron microscopy performed on as-formed whisker structures and cross-sections of the structures and underlying material. Individual samples are also investigated in an in situ manner in order to observe and monitor the growth behavior in real-time.

## 2. Materials and Methods

### 2.1. Material and Samples Preparation

Within this research, aluminum alloy EN AW 6026 with chemical composition (in wt. %) 0.70 Mg, 0.68 Si, 0.66 Bi, 0.59 Mn, 0.34 Pb, 0.30 Cu and 0.27 Fe (rest is Al), provided by Impol d.o.o., Slovenia, was used. The material was cut into 1 cm × 1 cm × 10 cm cuboids that were thermally processed using a standard procedure (homogenization) performed at 570 °C for 1 h and afterwards quenched in water. In the final treatment step, the samples were separated into 3 groups. The first group was not treated further. The second and third groups were exposed to deep cryogenic treatment (DCT) by immersing the samples in liquid nitrogen for 24 h and 48 h, respectively. This process allows the application of uniform shrinkage pressure onto the material, which in turn modifies the internal stress state of the material. As shown by different investigations, DCT reduces tensile residual stresses or even changes them into a compressive type [41,42,43,44]. For the whisker preparation, all samples were cut, ground down to 4000 grit paper and polished with 3 μm and 1 μm diamond suspension. The surface was finished with colloidal silica (25–40 nm particle size, 15 N load, 3 min polishing time). To guarantee identical surface preparation conditions and exposure to the different environments, the differently treated samples were embedded in the same resin holder (1 of each per resin holder) and metallographically prepared simultaneously. Immediately after surface preparation, the resin-embedded samples were exposed to different environments for 24 h in order to induce sufficient whisker growth on the sample surface; no etching was performed on the samples. Directly after the exposure, the samples were investigated with optical microscopy, scanning electron microscopy and transmission electron microscopy.

### 2.2. Methods

#### 2.2.1. Optical Microscopy

The Optical microscope Zeiss Axio Imager, Z2m, Carl Zeiss AG, Oberkochen, Germany, was used for the observation of the temporal evolution of whisker structures after exposure to different environments. The screening of the samples was performed directly after the exposure and then every day for the next 7 days. To visualize and separate the whisker structures from the matrix and other material phases, polarized light was used, which utilizes the different reflectance of the whiskers due to their material construct and non-planar structure (example provided in Supplementary Figure S1).

#### 2.2.2. Scanning Electron Microscopy

The sample surface and exerted structures were imaged with the Zeiss CrossBeam 550 (Carl Zeiss AG, Oberkochen, Germany) dual-beam electron microscope. Cross-sections of the selected specimens were performed by Ga+ focused ion beam (FIB) machining, operating the beam at 30 keV energy and currents ranging from 3 nA to 100 pA. Energy dispersive spectroscopy (EDS) was used to measure the chemical composition of the specimen using an OctaneElite EDS detector (EDAX, Mahwah, NJ, USA).

For the imaging of phases in the samples (whiskers) in a cross-sectional manner with the use of a focused ion beam—scanning electron microscope (FIB-SEM) Helios Nanolab 650 FEI, Hillsboro, OR, USA, at the Center of Excellence in Nanoscience and Nanotechnology—Nanocenter (CENN—Nanocenter). Samples (lamellas) for (scanning) transmission electron microscopy (S)TEM were also prepared with FIB. In the first step of the lamella preparation, samples were protected with a 300 nm thick electron deposited Pt layer and an additional 2.5 μm thick ion deposited Pt layer, which were deposited on top of each other at the selected ion acceleration voltages/beam currents of 20 kV/1.6 nA and 30 kV/0.4 nA, respectively. In the second step, lamellas were extracted from the sample and transformed into the Cu TEM grids with the use of an OmniProbe 200 micromanipulator. The third step comprised of thinning the lamellas from the initial thickness of 2 μm to the electron transparency (thickness of ≈30 nm). In the final step, the lamellas were cleaned with low kV ions/low beam current (0.5 kV/50 pA) for 1 min on each side, enabling the removal of the amorphous residue and Ga artifacts.

#### 2.2.3. Transmission Electron Microscopy

The cross-sectional samples were also investigated with probe Cs corrected scanning transmission electron microscope (STEM), Jeol ARM 200 CF, Jeol, Tokyo, Japan, equipped with a high-brightness cold field emission gun (CFEG) operating at 200 kV. Quali- and quantitative elemental chemical analyses were performed with energy dispersive X-ray spectroscopy using a Jeol Centurio wide-area Silicon Drift Detector (SDD) system and Electron Energy-Loss Spectroscopy (EELS) Gatan GIF Quantum ER Dual-EELS system.

Additionally, to the use of a dedicated STEM instrument, lamellas were also imaged and analyzed in a FIB using a STEM detector via selected techniques: high angle annular dark field ((H)AADF), dark-field (DF) and bright field (BF). The chemical composition of phases in the samples was determined in situ using Oxford Instruments X-max Silicon Drift Detector Energy Dispersive X-Ray Spectrometer (SDD EDS). The distribution of different phases was determined with EDS mapping.

#### 2.2.4. Vacuum Atmosphere

A stainless-steel vacuum chamber (in-house made) was used to store the sample in a controlled environment for 24 h. The chamber was evacuated down to about 10 mbar directly after inserting the sample. Mixtures of O_2_, N_2_ and CO_2_, each of 99.999% purity, were then introduced to the chamber, where pressure was continuously monitored with a capacitive diaphragm gauge with a measuring range of 1100 mbar. With this setup, desired ratios of O_2_ to N_2_ and CO_2_ to N_2_ were established in the chamber with an expanded relative uncertainty (with coverage factor k = 2) of 0.16%. Pressure change inside the chamber was below 3 mbar over the 24 h period. For the experiments, samples were exposed to atmospheres of 10% O_2_ and 90% N_2_, 20% O_2_ and 80% N_2_ and 40% O_2_ and 60% N_2_. Additionally, one experiment was conducted in an atmosphere of 95% CO_2_ and 5% N_2_. For all experiments, total pressure inside the chamber was 1000 mbar. For the varying moisture experiments, the ambient atmosphere was used, which is handled as an approximation to the 20% O_2_ condition. Stable relative humidity was achieved by introducing a Petri dish filled with a saturated salt solution to the chamber. A small battery-powered fan was used to ensure homogenous humidity across the whole chamber. The salt solutions used in the experiment and the resulting relative humidity are summarized in Table 1. Relative humidity was taken from the work of Wexler et al. (1954) [45].

## 3. Results and Discussion

### 3.1. Atmosphere Condition Dependency

**Figure 1:**
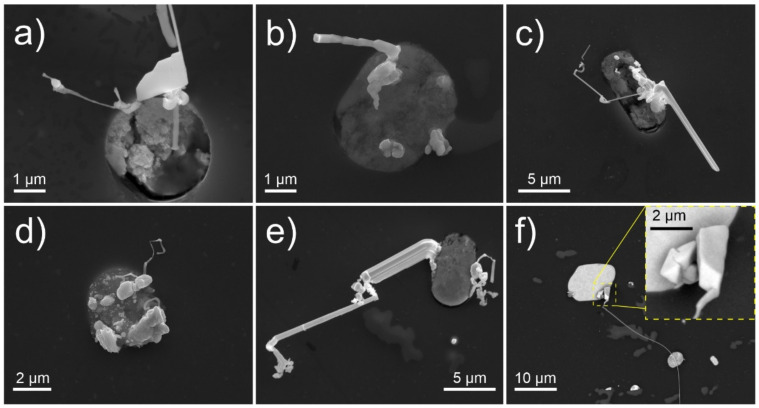
Secondary electron micrographs depicting various possible morphologies of Pb whiskers after 7 days of growth in ambient conditions. (**a**) widening plate-like sections, (**b**) segmented growth, (**c**) widening whisker base with multiple outgrowths, (**d**) blob formations, (**e**) branching and growth redirection and (**f**) cuboidal formations.

The microscopy observations indicate a variety of Pb whisker formations in both length and morphology that grow from the Bi-Mg-Pb pools. Generally, all samples display individual whiskers that reach lengths over 100 µm after 1 week of storage. Nevertheless, a majority of whiskers and hillocks display shorter lengths and, with it, increased occurrence. Additionally, these formations often display complex growth behavior and unique features such as widening plate-like sections (Figure 1a), segmented growth (Figure 1b), widening whisker base with multiple outgrowths (Figure 1c), blob formations (Figure 1d), branching and growth redirection (Figure 1e), as well as cuboidal formations (Figure 1f). The different morphologies indicate a complex growth mechanism that seems to depend on the conditions under which the whisker grows. In order to elucidate this dependency, systematic screening of the samples under different environmental conditions after 24 h of exposure was performed. Additionally, the samples were also prepared with different internal stress states in order to verify the effect of residual stresses on the whisker growth (see Section 2.2 for more details).

**Figure 2:**
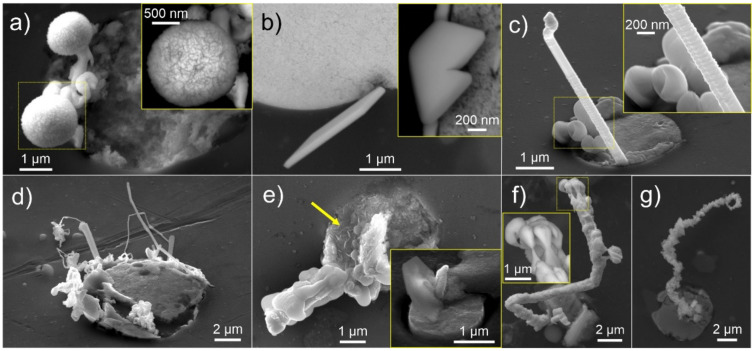
Secondary electron and back-scattering electron micrographs depicting typical examples of (**a**) bubbles displaying cotton ball-like morphology, (**b**) plates and facets, (**c**) whisker with enlargement indicating the growth serrations and (**d**) groupings constructed of several short whiskers intertwined with blob formations. (**e**) Typical hydroxide plate-like formations (marked with yellow arrow) formed on samples in an environment with high humidity. In (**f**,**g**), the occurring contorted whisker morphologies after 7 days of exposure to a high-humidity environment and high-oxygen environment are presented, respectively.

The microstructural investigation of the surfaces after 24 h in different environments (varying oxygen and humidity of atmosphere) yielded different predominance of specific types of growths. For further statistical analysis and interpretation of the significance of individual growths, they were classified into four major groups: bubbles and blobs, plates and facets, whiskers (subdivided by length into above and below 1 µm) and groupings. The examples of the individual formations are provided in Figure 2a–d. The exposure to different oxygen content yielded increased bubbles and blobs formation with increased oxygen content and reduced whisker and plate formation. On the other hand, increased humidity resulted in relatively unchanged whisker formation, except for 75% humidity and above, under which small plate-like features emerged (Figure 2e). This is a consequence of hydroxide formation on the exerted Pb material, which is induced by the high moisture content and results in structures that morphologically resemble the desert rose and are also typical of other metallic hydroxides [46]. Interestingly, the humid environment prevents the formation of faceted structures at lower oxygen content as well as increases the whisker formation compared to the dry condition with 20% oxygen content. The results suggest that the moisture contributes to the stress development in the system but at the same time, also causes the formation of hydroxides that changes the whisker morphology as well as the growth behavior. Further analysis with extended exposure of the samples for up to 7 days in a moist environment revealed that the whisker structures develop over time a thin layer, consisting mostly of C, O and traces of Pb, suggesting a carbonate form (see Supplementary Figure S2). The layer can cover the whole whisker length, but it is mainly reserved for thin whiskers (up to around 500 nm diameter). Thicker whiskers and segments either remain unaffected by the humid environment or display, in some instances, a melting-like behavior, which can also cause the alteration of the whisker form (see Supplementary Figure S3). In specific cases, the protruding whiskers display remolding caused by leaching that morphologically resembles stalagmite-like formations (Figure 2f). Such features indicate that the ionic exchange of the Pb must occur from the core of the whisker towards the hydroxide-formed surface of the whisker. This can also lead to the dissolvement of the whisker formation on a local level (see Supplementary Figure S3). In the case of a high-oxygen environment, the whisker structures display a sponge-like formation (examples in Figure 2g and Figure S4a). Based on the high oxygen content of the whisker surface (average of 63 atm. %), the structures results from the oxidation of the Pb into PbO_2_, which expands the volume (PbO_2_ has approximately 2g/cm^3^ lower density than Pb) of the whisker. The higher oxidation activation into PbO_2_ with the increased partial pressure of oxygen also goes in hand with the theoretical thermodynamic evaluation of the Pb-O system [47].

By combining high oxidation and high humidity on a local level, the formation of both separate structures was achievable, at which the humidity formed structures dominated over the oxidation forms. Additionally, the combination led to the emergence of crystallite florets that formed over the Bi-Mg-Pb solid pools (see Supplementary Figure S4b). Local EDS mappings reveal that the crystallites are enriched with O, Mg and C, corresponding to magnesium hydroxide crystals growing from a magnesium carbonate base (Supplementary Figure S4c). This indicates that, additionally to the interaction with Bi, magnesium also contributes to the oxidation and pressure build-up within the solid pools when sufficient humidity is present. Surprisingly, the formation of magnesium species did not interfere with the formation of Pb whiskers, as the two structures seem to form in a separate manner.

Due to the high carbon concentration of emerging structures, the material was exposed to dry conditions with 95% CO_2_ in order to test the whisker structure formation in a non-oxygen holding environment and to assess the possible relation of CO_2_ interaction with the whisker structures. In this case, the whisker emersion is not considerably altered from the low oxygen cases, indicating a negligible effect of pure CO_2_ on the formation of whiskers. Interestingly, the whisker structures are predominantly even more faceted and display generally smoother whisker structures, which suggest a relation to the partial oxidation of the whisker surface as well as the surface of the solid pools.

### 3.2. Statistical Analysis of Whisker Growth

**Figure 3:**
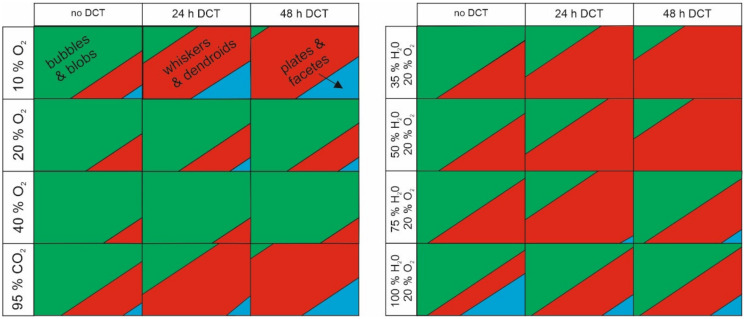
Morphological phase diagram depicting the individual fraction of specific growth groupings in dependency of the stored conditions and treatment procedure.

Based on the thorough investigation of the samples under different conditions, a phase map (Figure 3) was constructed to evaluate the emergence of different morphologies and their dependency on the storage conditions.

The phase map only displays the relative ratio of the different structures, whereas the actual change in density of the whisker and hillock formations will be covered in further steps of the statistical analysis. The phase map clearly shows that the most effective condition for forming whiskers is a low concentration of oxygen, as a high concentration of oxygen reduces the formation of pure whiskers and degrades their structures to porous formations and blobs. In retrospect, low oxygen content or the missing presence of oxygen allows a higher portion of the formed whiskers in the plate-like or facet forms. Furthermore, the lower oxygen content results in the preferential formation of faceted hillocks and faceted bubbles. On the other hand, the humidity generally does not affect it as strongly as oxygen content but still slightly reduces the formation of whiskers with higher humidity. Nevertheless, with high humidity, the development of plate-like whiskers and facets are promoted, which can be advantageous for morphologically constrained whisker growth. In relation to the state of the material, the increased exposure to DCT corresponded to a higher fraction of whiskers to other structures, regardless of the stored environment. This indicates that the material’s internal stress state (increased compression character) has a predominant role in the morphological development of the whiskers.

For designing and selecting the most optimal combination of experimental parameters for whisker growth and morphology modification, a Taguchi model of testing was developed. For the model, three levels were assigned to each factor, which was selected based on the feasibility of experimental preparation and coverage of measurement data in terms of whisker counting and morphology grouping. The used descriptive factors and levels via the Taguchi method are presented in Table 2, which results in only 12 experiments required to assess the general correlations and interdependencies of the selected factors. The exact combinations of required experimental conditions are presented in Supplementary Table S1. The three selected levels should represent reasonable extremes for each of the selected factors (oxygen rate, humidity level and DCT treatment). Together 24 samples were used (2 for each condition) and measured at 5 different locations (sampling area of 500 × 500 µm) per sample to obtain the most statistical representative data. The measured values are provided in Supplementary Table S2.

Using F factor ANOVA analysis of the measured data (ful analysis data provided in Supplementary Table S3), the contribution of individual factors as well as the cross-coupling of individual factors is assessed. The impact of individual factors and their doublet dependencies from the perspective of whisker density (number per area) and maximum whisker length are presented in Table 3 and Table 4, respectively. The model show that oxygen level and DCT have the most dominating effect on the whisker density on their own, whereas the combination of both oxygen and humidity had the highest impact on the whisker density in the sense of doublet combinations. To note, the contribution of oxygen is negative, whereas the other factors and combinations have a positive contribution to the whisker density. This is especially interesting from the point of the simultaneous contribution of humidity and oxygen, which translates to a positive contribution with lower oxygen content and intermediate to high humidity.

Similarly, the most contributing factor to whisker length is also the DCT and oxygen level. However, in this case, DCT dominates the contribution with over 60% weight. The oxygen contribution is also negative for the whisker length, but the combination with humidity results in a strong positive influence on the whisker length.

Based on all obtained results and relations, the most optimal environment to obtain long whiskers for further applications is an environment with 20% oxygen, 35% humidity level and 48 h of DCT. However, the interesting observation was that despite the absence of oxygen in the presence of CO_2_ and a moderate level of humidity, similarly long whiskers can be induced for the 48 h DCT sample. The possible reason for this is the strong contribution of the internal stresses of the material and the lack of oxidation of the pool surface that can obstruct the whisker growth. Furthermore, it is possible that the combination of the CO_2_-H_2_O mechanism, as observed by Both and Cheung 2019 [48], could potentially contribute to whisker growth, which will be discussed further in the following sections.

To understand the whisker growth tendencies with time and effect of internal stress, a temporal investigation of the samples was also performed with a one-way ANOVA analysis. The results, presented in statistical descriptive data, are given in Table 5 and Table 6. The results of ANOVA for the temporal dependency of whisker growth show that bubbles and groups dominate the sample surfaces (>200), and at the same time, the maximum (set as a threshold for counting) is already achieved by the second or third day, accordingly. The only significant difference between groups is for whisker type of length > 1 µm, which is associated with the strong initial burst of whisker growth that later on relaxes into a continuous eruption of whiskers with time. Furthermore, the results of ANOVA for the treatment (with or without DCT and 24/48 h DCT) dependency of the type of whisker growth showed that the whiskers with length > 1 µm and whiskers with length < 1 µm are most commonly present in 24 h DCT samples, whereas bubble and group types are present equally in all three treatments (no DCT, 24 h DCT and 48 h DCT). Additionally, the temporal dependency in relation to the presence of DCT provided a statistically significant difference between groups, which also indicates a similar relationship of whisker, bubble and groups formation with time for each group (similar ANOVA factor). In order to decouple the influence of the two dependent variables, time and treatment, an additional MANOVA was performed. The Wilks’ Lambda test provided time-variable *p* = 0.00 (*p* < 0.05), which additionally confirms that interaction is significant, meaning that time influences the formation of different morphologies, whereas for the treatment variable, the Wilks’ Lambda test showed (*p* = 0.757), indicating that no interaction is statistically significant (*p* > 0.05) and that the temporal whisker development does not change with relation to the different treatment.

### 3.3. Cross-Sectional SEM, TEM and STEM Whisker Analysis

With previous data, a general understanding of the effect of the storage environment on the development of different whisker morphologies was attained. However, the effect on the microstructural and surface structure of the whiskers needs to be resolved. For this reason, a series of cross-sectional investigations were carried out on selected whiskers to give insight into the structural development of the different morphologies.

**Figure 4:**
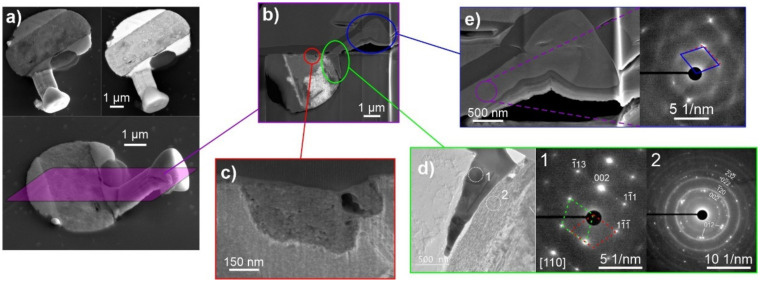
(**a**) Secondary electron and back-scattering electron images of the selected whisker and solid pool with the marked area through which the cross-section was performed. (**b**) Scanning electron microscopy micrograph of the cross-section. (**c**) Enlargement of the separated Bi regions formed within the Mg-Bi regions. (**d**) Enlargement of the Pb region situated on the edge of Bi-Mg region with local selected area electron diffraction (SAED) patterns corresponding to the polycrystalline Pb region (marked as 1) and polycrystalline Bi_2_Mg_3_ phase formed within a nanocrystalline Bi matrix (marked as 2). (**e**) Enlargement of the conjoined region of the whisker and faceted hillock with corresponding SAED depicting a single-crystalline Pb constructed of misoriented sub-domains.

**Figure 5:**
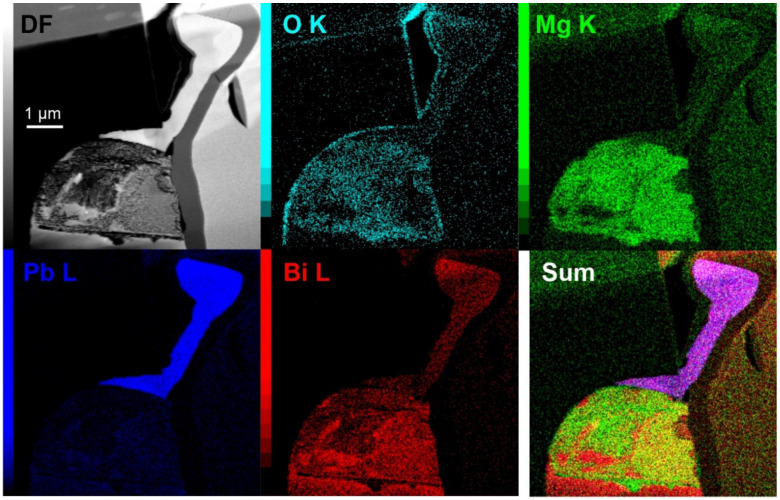
Dark-field (DF) image of the cross-section structure from Figure 4 with corresponding energy dispersive X-ray spectroscopy maps of selected chemical elements.

The first cross-sectional analysis, presented in Figure 4, for a sample stored with 20% oxygen, 35% humidity level and 48 h of DCT gives an example of a conjoined whisker structure with prior-formed faceted hillock from a pure separated phase (Figure 4a). The solid pool shows a clear phase separation into pure Bi (Figure 4c), Pb (Figure 4d,e), separated by an Mg-Bi region. The complementary EDS maps (Figure 5) indicate that the intermediate Bi-Mg region displays the strongest oxidation preference and that the near-surface Bi phases (Figure 4c) are also oxidated. The pure Bi and Pb regions on the left- and right-hand side, as well as the whisker, have a negligible oxygen signal, indicating that these phases remain in pure metallic form, which correlates well with the strong signal from the back-scattering electron image (Figure 4a) and local EDS maps (see also Supplementary Figure S5). Furthermore, the pure metallic form of Pb is confirmed by selected area electron diffraction (SAED), presented in Figure 4d,e. The electron images and EDS maps confirm the oxidation of the whole Bi-Mg region, which coincides with the enrichment with Mg. The SAED of the enriched Mg region indicates that the region is structured of Bi_2_Mg_3_ (COD ID 1010834) nanocrystals (marked indices of region 2 in Figure 4d) evenly distributed within a Bi matrix. The SAED display is stronger but also has more diffusive $00\bar{2}$ and $0\bar{2}2\;$rings and a diffraction halo in the short-range order, which indicates an amorphous to the nanocrystalline structure of the matrix. Diffraction pattern modelling indicates that the first diffusive ring corresponds to the pure Bi structure (COD ID 2310889), whereas the second one corresponds to the Bi oxide form Bi_2_O_3_ (COD ID 1010004). Due to the lack of higher-order index rings, which were only visible for the Bi_2_Mg_3_, the Bi matrix is considered to only have a short-range ordered structure with intermediate oxide formations.

The diffraction investigation of the whisker and faceted hillock reveals that the structures are crystallographically similar and confirm the single crystalline Pb structure throughout the entire structure. The SAED reveals a subset of misoriented subdomains of the structure, which goes in hand with the proposed whisker growth mode through oriented attachment, discussed in our previous paper [39]. The EDS maps indicate dominant Pb composition in the whisker structure but also shows a slight presence of Bi, which could be a result of the miscibility of up to 15 atm. % Bi in Pb, as expected by the Pb-Bi phase diagram. However, the Bi signal also shows a stronger signal leakage than Pb (see Supplementary Figure S6), which may cause an overshot of the actual Bi value. Interestingly, the Pb structure at the base of the whisker is different from the whisker and hillock (Figure 4d), which also shows a polycrystalline structure with twin structure features (see example lattice marking in SAED of Figure 4d). An in-depth analysis of the diffraction patterns reveals that the base shows a slight distortion of the Pb lattice (0.01 nm enlarged c-axis, 6° smaller angle α and 6° larger angle β), whereas the whisker displays a nearly ideal crystal structure of FCC Pb (COD ID 1011119). The difference is depicted in the SAED of Figure 4e, with the dashed lattice marking corresponding to the base structure and the full lattice marking to the structure of the whisker and hillock. This difference confirms that the stress induced by the surrounding oxidized material compresses the Pb material within the solid pool, which relaxes by forming the whisker and hillocks with a relaxed crystal structure.

**Figure 6:**
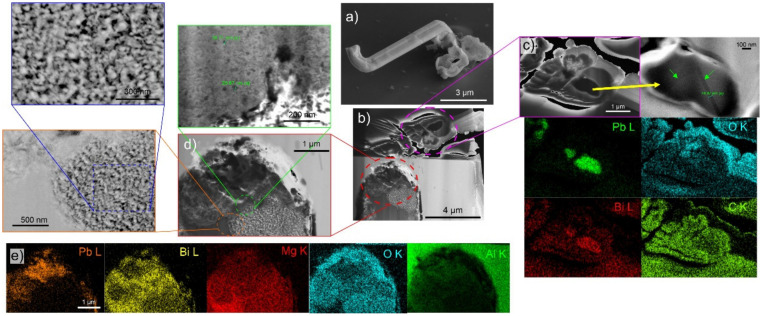
(**a**) secondary electron images of the selected whisker for cross-section analysis (**b**) Scanning electron microscopy micrograph of the cross-section. (**c**) Enlargement of the double-whisker structure with complementary high-angular dark-field image and energy dispersive X-ray spectroscopy (EDS) maps. (**d**) Enlargement of the upper portion of the solid pool with enlargements of individual regions. (**e**) EDS maps of region presented in (**d**).

The cross-sectional analysis of the whiskers formed under high humidity (Figure 6) displays a similar structure as seen beforehand under optimal whisker-growth conditions. The interesting aspect of the selected cross-section is the double-whisker structure, which can be clearly seen already before the cross-sectioning (Figure 6a). The cross-section (Figure 6b) clearly shows that the bulk of the whisker is formed from two separate whiskers that are conjoined. Additionally, the whisker is covered by a 50 nm thick layer (Figure 6c). The EDS analysis determines the layer to be enriched with O and C, suggesting a hydroxide or organic compound. Unfortunately, the obtained data could not provide more clear information about the structure of this layer but it still indicates that the high-moisture content contributes to the whisker growth only in a limited manner, without causing significant restructuring of the whisker. The high-angular dark-field imaging analysis (Figure 6c) indicated that the larger whisker is not, in fact, a single grain structure but is constructed of twins (marked by green arrows), which could be the reason for the change in whisker growth orientation and the double-whisker form. This also indicates that the growth structures are more intertwined between several growth seeds and that the growth is not a single-crystal linear growth phenomenon as anticipated by general whisker growth theories [3].

The solid pool of the whisker shows clearly the separation of the different phases as before, but in this case, the Pb and Bi regions are caught to be intermixed and provide a more realistic image of the state from which the whiskers are generally expected to grow. The Pb regions display a sponge-like structure and are surrounded by a Bi region below and an oxide layer above (Figure 6d,e). The Bi regions also hold small Bi_2_Mg_3_ crystallites, which are marked in the green enlargement. The Bi regions also display a sub-region which higher porosity (orange enlargement of Figure 6d), which is considered to be the main source of stress-build-up within the pool through oxidation. This exemplar structure of the pool also clearly shows the reason for the variable whisker growth and nucleation, as the different structures of the Bi regions can contribute differently to the rate of oxidation and volume expansion with the time that additionally drives the whisker growth.

**Figure 7:**
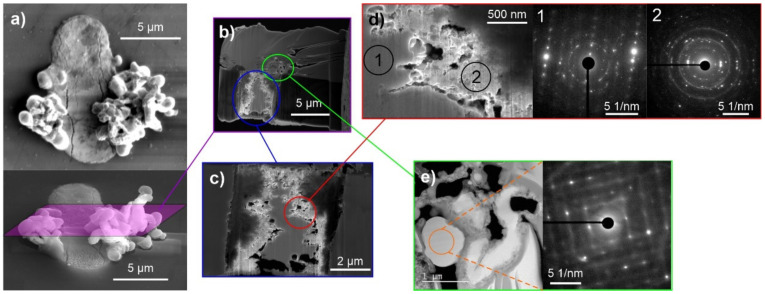
(**a**) secondary electron images of the selected whisker and solid pool with the marked area through which the cross-section was performed. (**b**) Scanning electron microscopy micrograph of the cross-section. (**c**) Enlargement of the Bi regions of the solid pool. (**d**) Enlargement depicting the area between the solid Bi (on left) and sponge-like Bi region (on right) with local selected area electron diffraction (SAED) patterns corresponding to the nanocrystalline Bi region (marked as 1) and polycrystalline Bi_2_Mg_3_ phase formed within a nanocrystalline/amorphous Bi matrix. (**e**) Enlargement of the whisker formations with corresponding SAED of the rounded feature, depicting a single-crystalline Bi constructed of misoriented sub-domains.

**Figure 8:**
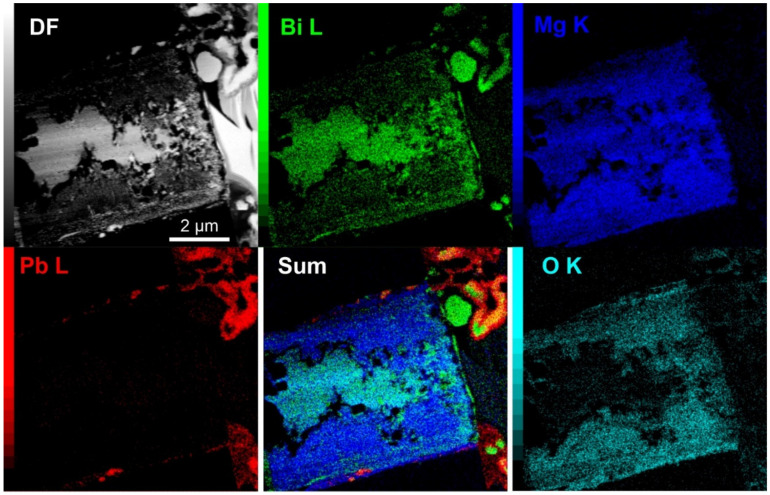
Dark-field (DF) image of the cross-section structure from Figure 7c with corresponding energy dispersive X-ray spectroscopy maps of selected chemical elements. The Sum is the combined color image of Bi L, Mg K and Pb L.

With higher oxygen content in the storage environment, the formed whisker structures were considerably unstable during FIB preparation, making the analysis difficult. For this reason, small structures in the initial whisker-development stage were cross-sectioned (Figure 7a). The cross-sectional analysis of these structures revealed a considerably different whisker growth compared to the previous two cases. The whiskers display a contorted form with many bulky-rounded structures. The cross-section reveals a considerably large Bi region with a small Pb region on the sides of the pool (see EDS analysis from Figure 8). Furthermore, the Bi region is subdivided into two regions, one displaying a solid Bi region situated in the middle, and a spongey structure, with high oxygen content, on the sides. In this case, it is clear that the presence of Mg does not influence the oxidation dynamics of the Bi regions since the Mg content is homogeneous across the whole Bi portion of the pool. The local SAED analysis confirms the nanocrystalline structure of the solid Bi portion and the presence of Bi_2_Mg_3_ nanocrystals as well as the oxide nanocrystals in the oxidized regions (marked regions 1 and 2 in Figure 7d) as observed with previous samples. The EDS analysis of the whiskers confirmed the Pb structure of the whiskers, but additionally, the whiskers displayed intermediate regions that corresponded to pure Bi structures (see also EDS analysis of enlargement in Supplementary Figure S7). Local SAED analysis showed that the Bi regions also display a crystalline structure that corresponds to the rhombohedral lattice structure of Bi. Interestingly, the Bi structures, even if they show a spherical shape, display a construct with misoriented sub-grains, as seen in Figure 7d, similarly as observed for the Pb whiskers. The EDS analysis also indicates that a thick oxide layer (100–200 nm) covers the whiskers, which is correlated only with the Pb signal, indicating that the Pb portions of the whiskers tend to oxidize faster than the Bi portions.

The analyses of the structures in high oxygen environment indicate that the high oxygen content induces the eruption of Bi material from the solid pools, possibly due to the faster oxidation of the pool material. As a result, the whiskers are contaminated with Bi, which causes the reduction of the pressure onto the Pb regions as well as forms the distortions of the whiskers due to the interchanging reformation of the crystal structure. Additionally, the increased oxygen presence causes the oxidation of the Pb whisker material, which causes a general decay of the whisker over time (as seen from previous SEM images).

**Figure 9:**
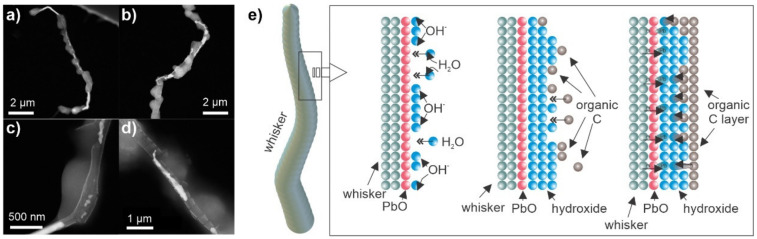
(**a**–**d**) Dark-field transmission electron micrographs of whiskers extracted from the sample after 7 days storage in high-oxygen and high-moisture environment. (**e**) Schematic presentation of the oxidation-hydration of the whisker surface and later enrichment with ambient organic carbon.

In order to understand the decay of the whiskers with exposure of the samples to a high-oxygen and high-moisture environment and the formation of the carbonate structure, the whiskers after 7 days of exposure were collected from the sample surface and investigated using TEM. Whiskers display a complex distorted structure (Figure 9a,b). The complementary EDS analysis (Supplementary Figure S8) confirms the simultaneous presence of both Bi and Pb in the whiskers, at which the Bi regions seem to be unaffected, whereas the Pb portions of the whiskers display a strong decaying behavior. The Pb regions are correlated to be overgrown structures developed from the leaching of the Pb material from the whisker structure, which is observed by the remnant silhouette of the prior whisker form (Figure 9c). Furthermore, the structures seem to form deeply from the Pb portions of the whiskers and cause breaking of the whisker structure by segmentation and outburst of the whisker silhouette (Figure 9d) due to expanding volume. The erupting structures are analyzed to be highly enriched with C with a small presence of Pb, without any strong presence of O, except for the edges of the whisker silhouettes. To note, the presence of Bi is still considerably lower compared to Pb in the whisker structures, despite the leaching of Pb. The local EDS analysis indicates roughly a 30% fraction of Bi in the whisker (exemplar EDS spectrum in Supplementary Figure S9).

We postulate that the additionally formed structures result from the formation of a covering film of oxides and hydroxides that allows the oxidation and leaching of the Pb whisker material. Due to the fragmented sub-structure of the whisker structure, the whisker holds a considerable number of local defects and low-angle grain boundaries, which allows faster leaching and oxidation into the bulk of the whisker than what would normally be possible for a solid Pb material. This, in combination with the affinity of water to agglomerate into droplets (coupled effect of Plateau–Rayleigh instability and capillary forces [49]), results in the segmented blob formations that form randomly across the whisker length, similar to water dew on spider webs. With the hydration of the surface, the surface can attract ambient organic carbon, which is absorbed into the whisker structure. Over time the Pb material is ionically leached to the carbon-rich regions from the whisker bulk, at which the intermediated oxide layer acts as a membrane that allows selective diffusion of Pb-ions. As such, the system behaves similarly to the leaching process of metal-carbon catalyst synthesis [50], which creates a ligand-type metal ion dispersed structure within an organic framework. The individual steps of this phenomenon are also schematically presented in Figure 9e. With electron energy loss spectroscopy (EELS), the carbon state is determined to be dominantly present in sp2 form, insinuating a double bond in the form of C=C or C=O (Supplementary Figure S10). However, the latter seems to be less feasible as the oxygen content is considerably low in the dissolved whisker structures. Additionally, the EELS spectrum indicates a predominant amorphous carbon form. The data as a whole indicates the possibility to transform the erupted whiskers into a carbon-rich sponge structure, which can be an interesting form to produce rod-like catalysis material. However, the feasibility of using such structures is defined by their production in a controllable manner. This might be achievable by constructing arrays of artificial solid pools with a similar structure as found in the investigated material and inducing the whisker growth through externally applied mechanical stress, and in the end, oxidizing and hydrating the whiskers through storage in the proper environment.

### 3.4. Oxidization-Enhanced Whisker Growth

**Figure 10:**
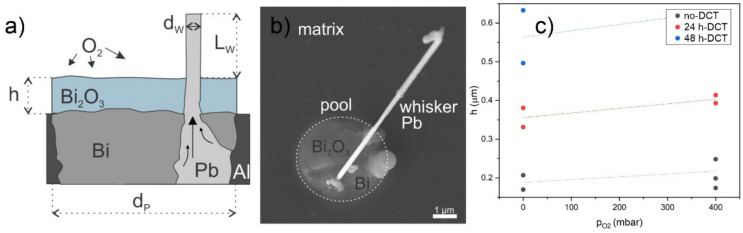
(**a**) Simplified schematic of the proposed mechanism of whisker growth (the Bi_2_O_3_ layer is intentionally overexaggerated for visualization purposes. (**b**) scanning electron microscopy image. (**c**) Thickness of the oxide layer Bi_2_O_3_, as calculated from average whisker length, is shown to be relatively strongly correlated to partial pressure of oxygen. The dashed lines graphically present the average trend between the quantities based on measured points.

Based on the microstructural and statistical evaluation of the different samples under different conditions, we propose that whisker growth is a consequence of stress inside the pool, which is additionally enhanced by the expansion of the Bi_2_O_3_ oxide layer. When Bi is oxidized, it expands by 60.4% (Cucka et al. [51] and Blower et al. [52]). Since the elastic modulus of Bi_2_O_3_ is considerably higher than that of pure Bi or Pb (nearly 2-fold and 4-fold, respectively [53,54,55]), the resulting stress is transferred preferentially to the interior of the pool (regions of Bi and Pb in Figure 10a) to accommodate the additional stress build-up.

Stress is released when the Pb region breaks through the oxide layer, resulting in a Pb whisker. Since only minor buckling of the Bi_2_O_3_ surface occurs, the volume of the ejected Pb (whisker volume *V_w_*) is considered to be equal to the volume difference Δ*V* resulting from the formation of the Bi_2_O_3_ layer. It thus follows that $\Delta V=\frac{1}{4}\pi d_{w}^{2}\times L$, where $d_{w}$ and L are the average whisker diameter and length, respectively, if the cross-section of the whisker is assumed to be circular. Volume after expansion V can be similarly estimated from the average surface $A_{P}\approx \frac{1}{4}\pi d_{P}^{2}$ of the pool and thickness of the oxide layer h, shown in Figure 10a. The relationship between h and L can then be derived from

(1) $$\frac{\Delta V}{V}=\frac{\pi d_{w}^{2}\times L}{V}=\frac{\pi d_{w}^{2}\times L}{\pi d_{P}^{2}\times h}={\left(\frac{d_{w}}{d_{M}}\right)}^{2}\frac{L}{h}$$

and then written as

(2) $$h={\left(\frac{d_{w}}{d_{P}}\right)}^{2}{\left(\frac{\Delta V}{V}\right)}^{-1}L.$$

Here, the ratio of whisker and pool diameter and whisker length can be determined from SEM images (example in Figure 10b). In our case, the ratio was found to be 0.05, and the average whisker lengths were taken for each sample individually from the data provided in Supplementary Table S2. The value of relative expansion ∆*V*⁄*V* is determined as 0.38 (Cucka et al. (1962) [51] and Blower et al. (1988) [52]). The thickness of the Bi_2_O_3_ layer is influenced by the partial pressure of oxygen surrounding the sample surface. Therefore, if the theory of oxidization-driven whisker growth is correct, the thickness of the oxide layer, calculated directly from whisker length and partial pressure of oxygen, should be correlated. We have taken the cases with RH = 0% from our set of measurements since the inclusion of H_2_O might complicate this behavior and oxidation dynamics. The calculated oxide thickness as a function of partial pressure of oxygen *p_O2_* is shown in Figure 10c. The data shows a correlation between higher oxidation thickness with higher *p_O2_*. There is a notable distinction between no-DCT and 24 h-DCT samples, so in order to determine the correlation, the two samples should be separated. The correlation factors of these data points were calculated to be 0.43 and 0.78 for no-DCT and 24 h-DCT samples, respectively. This relatively strong correlation for both samples supports the proposed mechanism of oxidization-driven whisker growth. The correlation of 24 h-DCT is stronger than for no-DCT, but the reasoning behind this is as of yet unknown and more research is needed to understand this relation.

### 3.5. In Situ Observation of Whisker Growth

Up to now, the dynamics of the whisker growth are addressed from a several-hour point of view, which is also covered also in our previous publication [39]. However, for an in-depth understanding of the whisker growth modes and behavior a more time-resolved investigation is required in the seconds-to minute regime. With this in mind, the whisker growth was monitored with SEM directly after 1 day of exposure of the samples to the different environments. Since the interest of these observations is to unravel the growth behavior, the whiskers are analyzed in their beginning state directly during or after initial nucleation. From the point of different storage environments, not many differences were observed in the whisker growth evolution, indicating that the initial stages of whisker growth depend on the stress-release effect and local morphological situation of the solid pools. This also infers that the development into the different morphologies occurs at a later stage when the initial hillock/whisker structure matures to a critical point, where the exerted mass and its reaction with the environment contributes to the morphological stability of the exerted structure.

**Figure 11:**
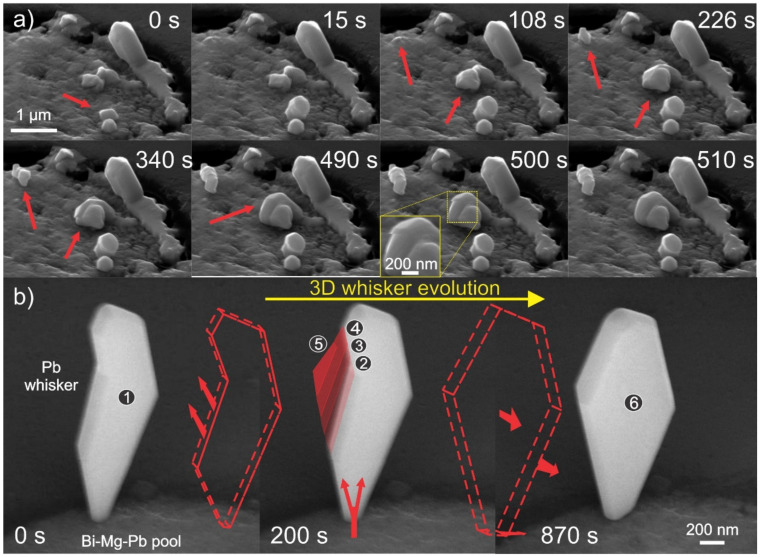
(**a**) image sequence of hillocks and initial whisker growth displaying sequential growth and morphology changes during the growth process. The red arrows indicate the regions of interest that change from image to image within the allotted time stamp. (**b**) image sequences of a faceted whisker growth displaying firstly a sectional growth from the initial state (marked as 1) that grows in segments (marked 2–5). The whisker afterwards also shows broadening of the whole structure over time (marked as 6). The intermediate sketches depict the changes from figure to figure. The arrows in the middle image depict the flow of material into the whisker structure.

From the direct in situ SEM observations, the stepwise growth of the whisker segments is revealed. In Figure 11a, the sequential development of hillocks and initial whiskers indicate that the growth occurs in steps, and the growth extends from several tens to hundreds of nanometers within a few seconds to minutes. Furthermore, the evolution of the hillocks reveals a highly stochastic growth process, which can display extremely fast growth steps. The whole growth evolution in video form is provided in Supplementary Video S1. An example of the fast growth behavior is provided in the image of 500 s in Figure 11a. The change of the hillock from state 490 s to 510 s occurs exactly at the 500 s mark, which can be seen by the discontinuity in the scanning lines, which is also magnified and corresponds to a change that occurs within one scanning sequence. Since the scanning time of the whole image is set to 10 s, this correlates to a line scan time of 13 µs. The edge displacement is used to associate the same pixel position change, which results in a displacement of 30 nm. With the known line scan speed difference (2 × line scan time), the resulting growth velocity of the small segment corresponds to roughly 1.15 µm/s. These extremely fast growth speeds also correspond to the fast development of thin whiskers that are observed in this study (example provided in Supplementary Figure S11). The stepwise growth is considered to occur due to the eventual stress-induced material agglomeration at the subsurface of the whisker base, which is then released in a single material spurt once the local surface energy and adhesion to surrounding material are overpowered by the pressure build-up at the subsurface. It is postulated that the degree and amount of exerted material depend on the local pressure development and available material in the whisker reservoir below it. As can be seen from the evolution in Figure 11a, the segmented growth is not a result of the oxide confinement as the SEM observations are conducted in high vacuum conditions during which the nucleation and growth of exerted structures were observed. Additionally, the segmented growth explains the sub-domain structure of the whiskers, as seen in Figure 4e, as the stepwise growth of the whisker can result in a slight mismatch of the segments in terms of their crystallographic orientation. This is also further supported by the observed torsion of the grown structure in the upper-left portion of Figure 11a. Such behavior also explains the occasional formation of serrations (see Figure 2c) in certain whiskers, which are a result of the widening and narrowing of the growth segments based on the set time required for eruption and interplay of surface energy and confinement by surrounding oxides [24].

Another example of the interesting segmented growth behavior is presented in Figure 11b. In this case, the growth of the more faceted whisker form is monitored, which reveals that growth can occur not only in a stepwise motion along the length but also along the width or portion of the structure. As can be seen from the image sequence and Supplementary Video S2, the structure grows in a manner similar to that of dislocation stepping. The phenomenon is much more fascinating when the growth base dimensions are compared to the dimension of the structure, which follows the extremely high material throughput through the narrow base in order to accommodate such large changes in the structure. The stepwise motion depicted in the middle image of Figure 11b supports the agglomeration of the material within the structure and the relaxation of the built-up pressure again through the extension of the structure. Interestingly, once the structure reaches an energetically preferred form through faceting, the whole structure enlarges in a monotonic fashion in all directions. Such behavior provides evidence of the continuous flow of the material into the erupted structure and that the surface energy plays an important role in the preferred growth direction and morphology, which is also confirmed by other authors on Sn-whisker growth [33]. The volumetric difference analysis indicates that the stepwise growth corresponds to a volume change of 0.023 µm^3^ in 200 s and the monotonic growth corresponds to a 0.05 µm^3^ broadening within the 670 s, corresponding to 115 × 10^−6^ to 75 × 10^−6^ µm^3^/s volume growth, respectively. The evaluated growth velocities correlate well to expected whisker growth rates determined from modelling by Chason et al. [8].

### 3.6. Discussion on Whisker Nucleation and Growth

Both previous examples provide evidence of the stepwise growth activation and behavior of the whiskers, which is generally not described in previous literature. The commonly accepted theories indicate the growth to be a continuous flow and exchange of vacancies and atoms from higher to lower pressure zones and vice versa. However, from the experimental observations at hand, this is clearly not the case. The agglomeration behavior provides support to our proposed theory from a previous paper on oriented attachment growth mode [39], which allows the reformation of the whisker form and growth in a funnel-like morphology. Furthermore, the other proposed theories cannot explain the observed growth behavior as the diffusion of vacancies of atoms cannot occur beyond the opening of the whisker growth as the pressure change directions prevent overflow of the whisker material into the center of the erupting structure. As such, by the conventional theories, the only possible growth mode would be a bottom-up or bottom-side growth, which falls flat in explaining the observed growth behavior and morphologies that have been presented in this and our previous paper [39]. With our theory, the viscous-like behavior of the material inside the whisker allows its reformation and restructuring over time and with internal pressure changes. Furthermore, the oriented attachment growth mode gives way to explaining the transitioning restructuring of the whisker towards a single crystal structure (confirmed by previous observation [39]), which seems to develop from a polycrystalline structure as confirmed by TEM observations of the initial whiskers in their early stage. The theory also explains the local reforming under humidity and high-oxygen environments since the local misaligned structures provide a large number of defects and surfaces that can contribute to the higher reactivity and diffusivity of the whisker material with external triggers. Due to the possible reformation dynamics with such growth mode, the different erupted structure morphologies can be explained (ranging from plate structures with dissimilar bases, blocky spherical structures with evidential segmentations, faceted whiskers, smooth whiskers with enlarged base, segmented whisker formation to and whiskers with modified narrowing and broadening through its length without temporally modified whisker base). Examples of the different morphologies are provided in Supplementary Figure S11.

Fundamentally, such a conclusion gives insight into why the nucleation of whiskers can range from seconds to years as the build-up of the material at the near-surface zone is crucial and that a reservoir of the material has to exist beforehand in order to cause whisker formation. This goes well hand in hand with observation of whiskers induced in Sn materials, where the low-angle surface grains were discussed as the nucleation points for whiskers. However, these in-fact might be just agglomeration of compressed material that forms at specific points due to the locally lower surface energy, which later acts as a weak point for the whisker formation. As such, the diffusion theories are correct on how the material flows towards the weak points, but not from the perspective of how the whiskers nucleate. The agglomeration theory is also supported by the perspective of modelling from other researchers that have indicated that a local pressure zone must develop around the whisker for it to grow [8]. Mostly, this was associated with the pressure difference within the whole structure and described as some local changes caused by either material inhomogeneity, oxide development, intermetallic compounds, grain boundaries or other structural discontinuities [1,2,3]. However, also researchers pointed out that this made, in some cases, little sense when no such indications were found to exist around certain whiskers. Additionally, the proposed nucleation and growth theory provide a reason for the reduced whisker nucleation with increasing temperature [1,2,3]. It is proposed that with increasing temperature, the surface energy is reduced, and the diffusivity of the system is increased, which generally would cause a tendency to increase whisker nucleation. However, with the increase of temperature and the diffusivity of the material, the effect of other surrounding weaker points is also activated, causing a reflow and redistribution of the material to also the other weak points. As such, the “effective pressure” on a single local point with agglomerated material is reduced, which decreases the nucleation capability of the whisker from the specific location. As such, it is clear that the newly determined whisker growth resolved in the second timespan provides important clues as to how the whiskers nucleate, grow and reform at the initial stage, which is significant for whisker mitigation in other systems as well as controllable production for this selected system.

## 4. Conclusions

The influence of different environments in terms of oxygen content, humidity and gas composition was resolved in relation to the Pb whisker growth dynamics from Bi-Mg-Pb solid pools from an Al-alloy surface. From our experiments and observations, we conclude that:

1. Both increasing humidity and oxygen content of the storage environment reduces the whisker formation, which in all cases form in a single-crystal-like structure constructed of slightly misaligned subdomains.
2. With humidity, the morphology of whiskers is generally not strongly modified until very high humidity towards 75% and above is used. This is a result of the development of a thin oxidized layer that remodels at higher humidity due to ionic leaching through a covering water-rich layer.
3 With higher oxygen content, the oxidation of the whisker formations and surrounding material increases, leading to stronger confinement of the whiskers as well as the conversion of the metallic whiskers to an oxide form with a sponge-like and mechanically unstable structure. The high-oxygen environment induces additional ingestion of Bi into the whisker, resulting from higher pressure build-up through the stronger and faster oxidation of surrounding material.
4. Overall, with lower oxygen and humidity content as well as 95% CO_2_, the whiskers pertain to more faceted and smooth surfaces, but at the expense of lower nucleation rate and lower whisker density. With intermediate O_2_ (20%) and humidity (35%), the elongated rod-like whiskers exhibited the highest densities and maximal length.
5. The increasing compressive internal stresses through deep cryogenic treatment of the aluminum-alloy can enhance whisker formation as well as the maximal achievable length of whiskers by up to 3× and 7×, respectively.
6. Significant whisker reformation occurs under a high-humidity and high-oxygen environment with a longer exposure time of upwards of several days. Under these conditions, the simultaneous oxidation and hydration of the whisker surface occur, resulting in the formation of a carbon-rich sponge-like structure. The structure originates from the leaching of Pb to the surface due to the enrichment of the surface with carbon and hydroxides. The resulting sponge-like structure is a very promising formation for the development of high-surface holding materials for catalytic or sensor applications.
7. The first-time in situ measurements in the seconds time span provide the first direct evidence of the stochastic stepwise growth behavior of spontaneously grown metallic whiskers on the sub-micron scale. The growth bursts were determined to occur with growth velocities up to 1.15 µm/s and volumetric changes of 1 × 10^−4^ µm^3^/s. The in situ evaluated growth and reformation of the emerging structures indicates a more complex growth mechanism than expected from previous research and theories. With our previous proposed theory of oriented attachment assisted recombination of small crystallites, the stochastic nature of spontaneous whisker nucleation and growth is fundamentally explained as well as the formation of various morphologies that also depend on the local surface energy balance and confinement by oxidation and surrounding material.

## Figures and Tables

**Table 1:** Values of relative humidity (RH) based on introduced salt solution to the experimental chamber.

Salt Solution	RH
Pure H_2_O, saturated with MgCl_2_	33.3%
Pure H_2_O, saturated with NaCl	75.7%
Pure H_2_O	100%

**Table 2:** Factors and level description for Taguchi design testing method.

Factor	Level		
	1	2	3
A: oxygen rate	0% (A1)	20% (A2)	40% (A3)
B: humidity level	dry (0–5%) (B1)	35% (B2)	75% (B3)
C: DCT	0 h (C1)	24 h (C2)	48 h (C3)

**Table 3:** The most significant parameters for growth (number) of whiskers based on F factor of Taguchi method—ANOVA.

		Percentage Contribution (%)
A	Oxygen level	50.251
B	Humidity level	0.574
C	DCT	13.325
A × B	Oxygen rate × humidity level	20.436
A × C	Oxygen rate × DCT	5.560
B × C	Humidity level × DCT	9.827

**Table 4:** The most significant parameters for whisker length based on F factor of Taguchi method—ANOVA.

		Percentage Contribution (%)
A	Oxygen level	12.598
B	Humidity level	0.457
C	DCT	62.254
A × B	Oxygen rate × humidity level	0.985
A × C	Oxygen rate × DCT	14.021
B × C	Humidity level × DCT	8.685

**Table 5:** The time (5 h, 1 day, 2 days, 3 days, 4 days and 7 days) dependency on type of the whisker growth.

Type of Whisker	Mean	SD	Min	Max	Variance	F	ANOVA (*p* > 0.05)	Brown–Forsythe Test
Length > 1 µm	11.6	21.9	0 (3–7 days)	90 (1 day)	0.003	2.852	0.064	-
Length < 1 µm	108.1	57.1	16 (1 day)	211 (7 days)	0.037	35.044	0.000	0.002
Bubble	153.2	69.9	31 (1 day)	>200 (2–7 days)	0.001	51.408	0.000	-
Groups	133.9	76.8	20 (1 day)	>200 (3–7 days)	0.002	99.996	0.000	-

**Table 6:** The treatment (DCT = no, 24 h and 48 h) dependency on type of the whisker growth.

Type of Whisker	Mean	SD	Min	Max	Variance	F	ANOVA (*p* > 0.05)	Brown–Forsythe Test
Length >1 µm	11.6	21.9	0 (all)	90 (24 h DCT)	0.114	0.863	0.442	0.463
Length < 1 µm	108.1	57.1	9 (no DCT)	211 (24 h DCT)	0.788	0.127	0.881	0.881
Bubble	153.2	69.9	5 (no DCT)	>200 (all)	0.803	0.157	0.856	0.856
Groups	133.9	76.8	6 (no DCT)	>200 (all)	0.747	0.066	0.937	0.937

## Data Availability

The raw/processed data required to reproduce these findings cannot be shared at this time as the data also forms part of an ongoing study.

## Supplementary Materials

The following supporting information can be downloaded at: https://www.mdpi.com/article/10.3390/ma15072574/s1, Figure S1: Exemplar image of a whisker visualized with brightfield optical microscopy and polarized light microscopy; Figure S2: Secondary electron micrographs depicting examples of whiskers displaying a thin oxidation/carbonate layer; Figure S3: Secondary electron micrographs depicting the effect of local dissolvement of thin whiskers in high-humidity environment; Figure S4: Secondary electron micrographs and EDS maps of oxidated whisker formed under high-oxygen environment and floret structures formed under high-humidity environment; Figure S5: Dark-field transmission electron image with corresponding energy dispersive X-ray spectroscopy maps of the whisker border indicating no oxide formation under 20% O2 content and 35% humidity; Figure S6: Energy dispersive X-ray spectroscopy maps of the whisker formed under 20% O2 content and 35% humidity, depicting the higher signal leakage of Bi compared to Pb and the effective summarization reduction of Bi signal with proper color selection; Figure S7: Dark-field (DF) transmission electron image with corresponding energy dispersive X-ray spectroscopy maps of the contorted Pb- and Bi-holding whisker formed under high-oxygen environment; Figure S8: Energy dispersive X-ray spectroscopy maps of the contorted Pb- and Bi-holding whisker formed under high-oxygen environment; Figure S9: Example of energy dispersive X-ray spectroscopy spectra from a Pb- and Bi-holding whisker; Figure S10: Electron energy loss spectroscopy within the characteristic carbon range for individual whiskers with carbonous layer formed after 7-days exposure in high-oxygen and humidity environment; Figure S11: Scanning electron microscopy (SEM) images depicting various additional morhpolgies and growth of whiskers; Table S1: Possible interactions of selected factors based on Taguchi method design; Table S2: Experimental results for Taguchi method design for selected factors (oxygen rate, humidity rate and DCT); Table S3: Taguchi method initial ANOVA; Video S1: Video of hillocks and initial whisker growth obtained with scanning electron microscopy; Video S2: Video of sectional growth of faceted whisker and subsequent monotonic widening obtained with scanning electron microscopy.
