# Supplementary material for: Revealing the Pb Whisker Growth Mechanism from Al-Alloy Surface and Morphological Dependency on Material Stress and Growth Environment
Source: Materials (Basel). 2022 Mar 31;15(7):2574. doi: 10.3390/ma15072574 (PMC8999954; doi:10.3390/ma15072574)
Supplement: Supplementary file 1 [file materials-15-02574-s001.zip › materials-1654591-supplementary.pdf]

materials

MDPI

Article

Revealing the Pb Whisker Growth Mechanism from Al-Alloy Surface and Morphological Dependency on Material Stress and Growth Environment

Matic Jovičević-Klug [1,*], Tim Verbovšek [2], Patricia Jovičević-Klug [2,3,*], Barbara Šetina Batič [2], Bojan Ambrožič [4], Goran Dražić [3,5] and Bojan Podgornik [2,3]

[1] Max-Planck-Institute für Eisenforschung GmbH, Max-Planck-Straße 1, 40237 Düsseldorf, Germany
[2] Institute of Metals and Technology, Lepi Pot 11, 1000 Ljubljana, Slovenia; tim.verbovsek@imt.si (T.V.); barbara.setina@imt.si (B.Š.B.); bojan.podgornik@imt.si (B.P.)
[3] Jožef Stefan International Postgraduate School, Jamova Cesta 39, 1000 Ljubljana, Slovenia; goran.drazic@ki.si
[4] Center of Excellence in Nanoscience and Nanotechnology, Jamova Cesta 39, 1000 Ljubljana, Slovenia; bojan.ambrozic@ijs.si
[5] National Institute of Chemistry, Hajdrihova Ulica 19, 1000 Ljubljana, Slovenia
* Correspondence: m.jovicevic-klug@mpie.de (M.J.-K.); patricia.jovicevicklug@imt.si (P.J.-K.); Tel.: +49-2116-792-347 (M.J.-K.); +386-1470-1990 (P.J.-K.)

Citation: Jovičević-Klug, M.; Verbovšek, T.; Jovičević-Klug, P.; Šetina Batič, B.; Ambrožič, B.; Dražić, G.; Podgornik, B. Revealing the Pb Whisker Growth Mechanism from Al-Alloy Surface and Morphological Dependency on Material Stress and Growth Environment. *Materials* **2022**, *15*, 2574. https://doi.org/10.3390/ma15072574.

Received: 11 March 2022
Accepted: 28 March 2022
Published: 31 March 2022

Publisher's Note: MDPI stays neutral with regard to jurisdictional claims in published maps and institutional affiliations.

Copyright: © 2022 by the authors. Licensee MDPI, Basel, Switzerland. This article is an open access article distributed under the terms and conditions of the Creative Commons Attribution (CC BY) license (https://creativecommons.org/licenses/by/4.0/).

Supplementary data

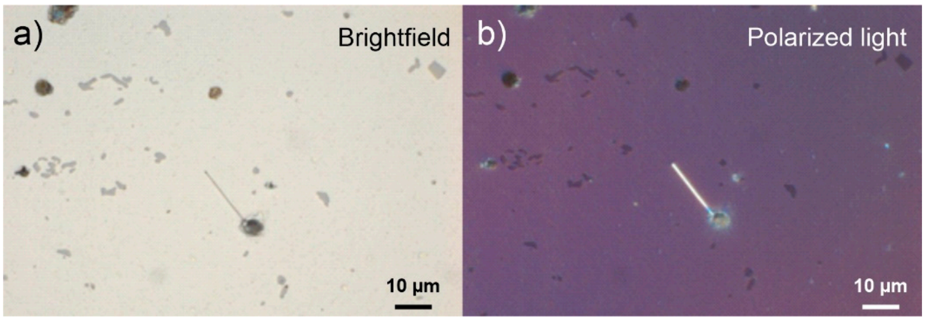

Figure S1. (**a**) Exemplar image of a whisker visualized with brightfield optical microscopy and (**b**) the same whisker imaged with polarized light microscopy for higher contrast separation of whiskers from matrix and other microstructural features.

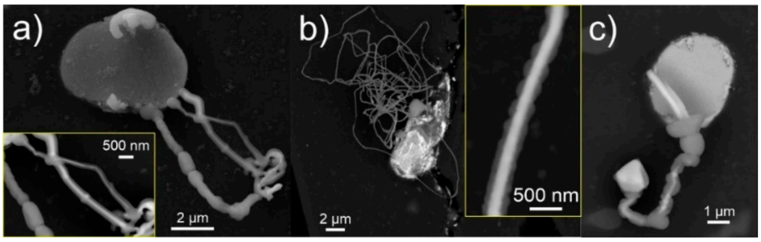

Figure S2. Secondary electron micrographs depicting examples of whiskers displaying a thin oxidation/carbonate layer. The oxidation can occur on (**a**) thin whiskers of short and straight form as well as on (**b**) thin whiskers of exceedingly long and contorted form. (**c**) The oxidation/carbonate layer is constricted to thinner portions of the whiskers, irrespectively of their position and temporal occurrence.

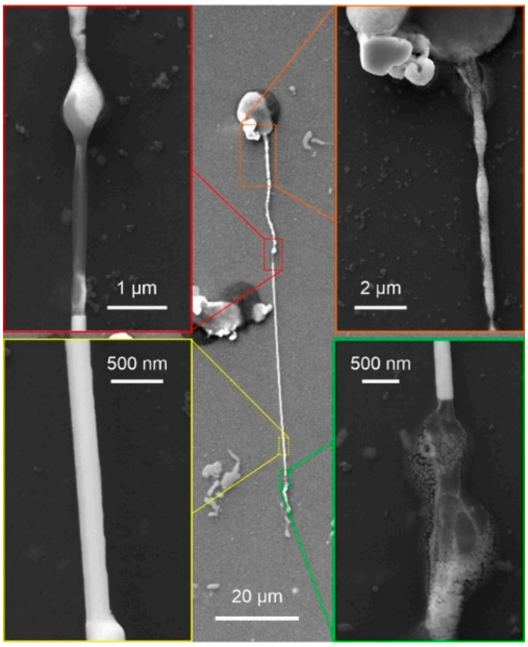

Figure S3. Secondary electron micrographs depicting the effect of local dissolvement of thin whiskers in high-humidity environment.

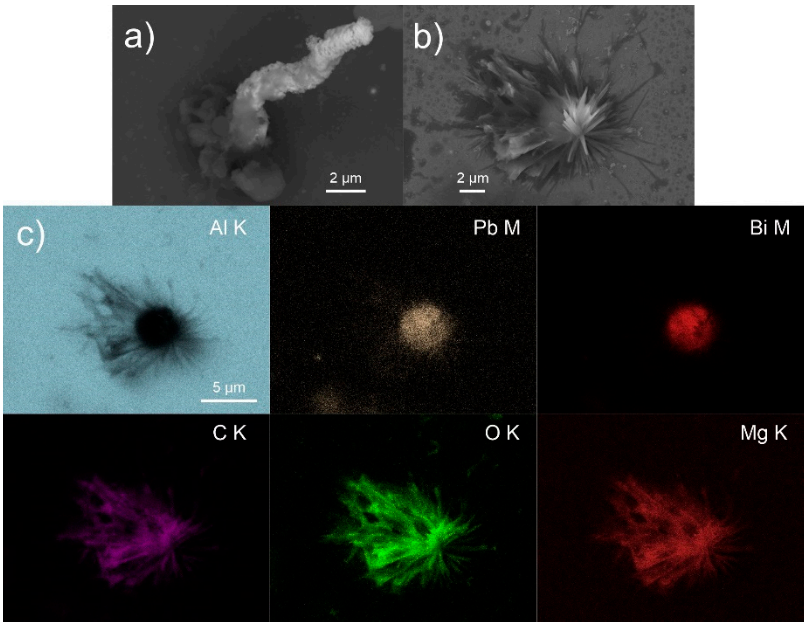

Figure S4. Secondary electron micrographs of (**a**) oxidated whisker formed under high-oxygen environment and (**b**) floret structures formed under high-humidity environment. (**c**) Energy dispersive X-ray spectroscopy maps of the floret structure presented in image (**b**).

Table S1. Possible interactions of selected factors based on Taguchi method design.

Exp. No.	Interactions
1	A3 × B1 × C2
2	A2 × B2 × C1
3	A1 × B1 × C2
4	A1 × B1 × C3
5	A3 × B1 × C1
6	A2 × B3 × C2
7	A2 × B2 × C2
8	A2 × B3 × C1
9	A2 × B3 × C3
10	A2 × B2 × C3
11	A1 × B1 × C1
12	A3 × B1 × C1

Table S2. Experimental results for Taguchi method design for selected factors (oxygen rate, humidity rate and DCT). The average longest whisker length was determined from averaging the length of the 10 longest whiskers found on each sample surface.

	Sample 1		Sample 2	
Exp. No.	Average Number of Whiskers After 7 Days	Average Longest Length of Whisker after 7 Days (μm)	Average Number of Whiskers after 7 Days	Average Longest Length of Whisker after 7 Days (μm)
1	77	100	75	95
2	142	160	135	186
3	118	80	98	92
4	170	120	185	153
5	102	60	98	48
6	212	270	222	305
7	187	280	179	324
8	114	110	121	99
9	224	350	235	386
10	211	430	198	498
11	85	50	93	41
12	43	60	38	42

Table S3. Taguchi method initial ANOVA. Using the principle of pooling up technique, with the insignificant factors, where the F value was below selected values. The orange-colored F values correspond to the selected values, whereas the gray-colored F values correspond to the disregarded values.

Exp. No.	SD number	Between-component variance	d.f. number	Homogeneity of variances number	F number (x10^4)	SD length	Between-Component variance	d.f. length	Homogeneity of variances length	F length (x10^4)
1	1.05	1.9	1	0.886	3.751	3.5	12.5	1	1.000	25.000
2	3.69	24.5	1	1.000	49.000	13.70	338.0	1	1.000	676.000
3	10.54	200.0	1	1.000	40.000	6.33	72.0	1	1.000	144.000
4	7.91	112.5	1	1.000	225.000	17.39	544.5	1	1.000	108.900
5	2.15	8.3	1	0.044	0.276	6.33	72.0	1	1.000	144.000
6	5.27	50.0	1	1.000	100.000	18.45	612.5	1	1.000	12.200
7	4.22	32.0	1	1.000	64.000	23.19	968.0	1	1.000	19.300
8	3.69	24.5	1	1.000	49.000	5.79	60.5	1	1.000	121.000
9	5.79	60.5	1	1.000	121.000	18.97	648.0	1	1.000	12.900
10	6.85	84.5	1	1.000	169.000	35.84	2312.0	1	1.000	462.400
11	4.22	32.0	1	1.000	64.000	4.74	40.5	1	1.000	81.000
12	2.64	12.5	1	1.000	25.000	9.49	162.0	1	1.000	324.000

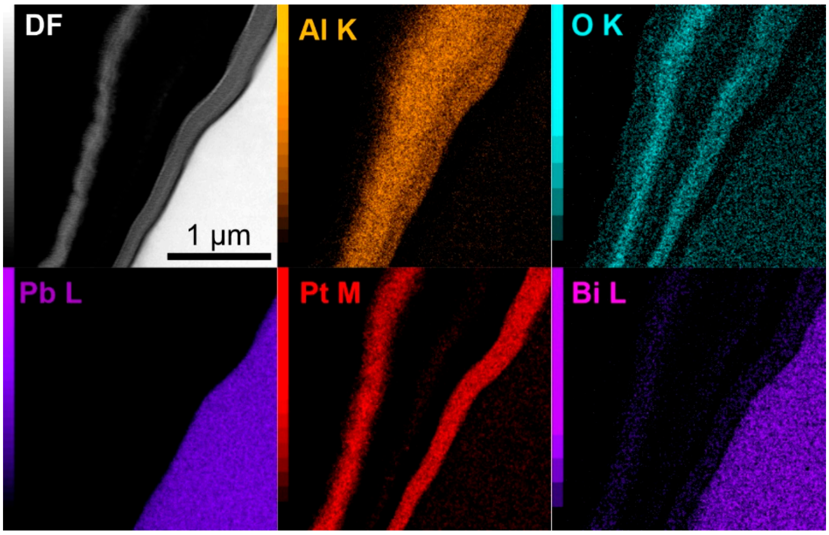

Figure S5. Dark-field (DF) transmission electron image with corresponding energy dispersive X-ray spectroscopy maps of the whisker border indicating no oxide formation under 20% O_2 content and 35% humidity.

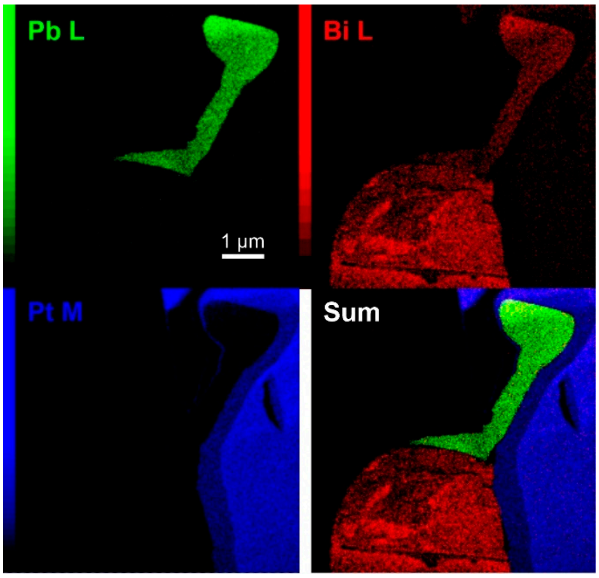

Figure S6. Energy dispersive X-ray spectroscopy maps of the whisker formed under 20% O_2 content and 35% humidity, depicting the higher signal leakage of Bi compared to Pb and the effective summarization reduction of Bi signal with proper color selection.

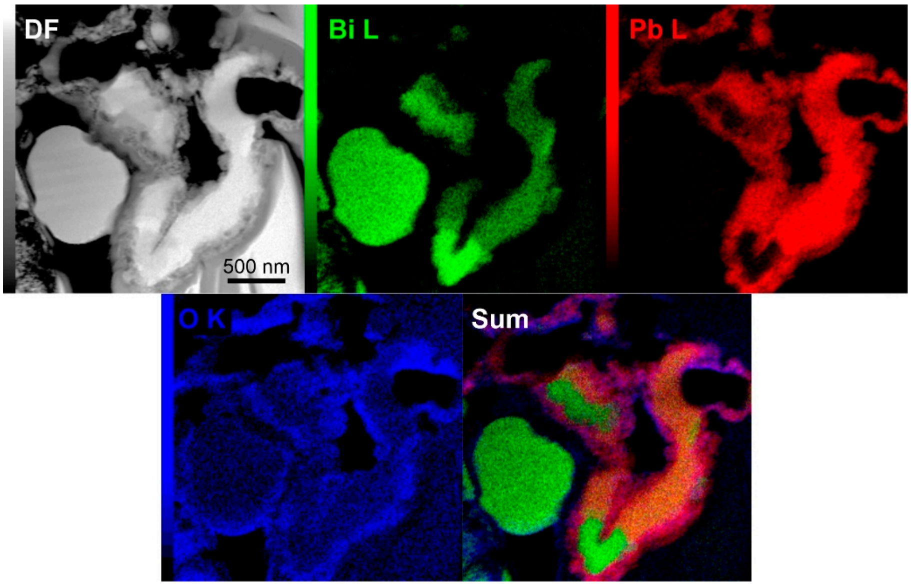

Figure S7. Dark-field (DF) transmission electron image with corresponding energy dispersive X-ray spectroscopy maps of the contorted Pb- and Bi-holding whisker formed under high-oxygen environment.

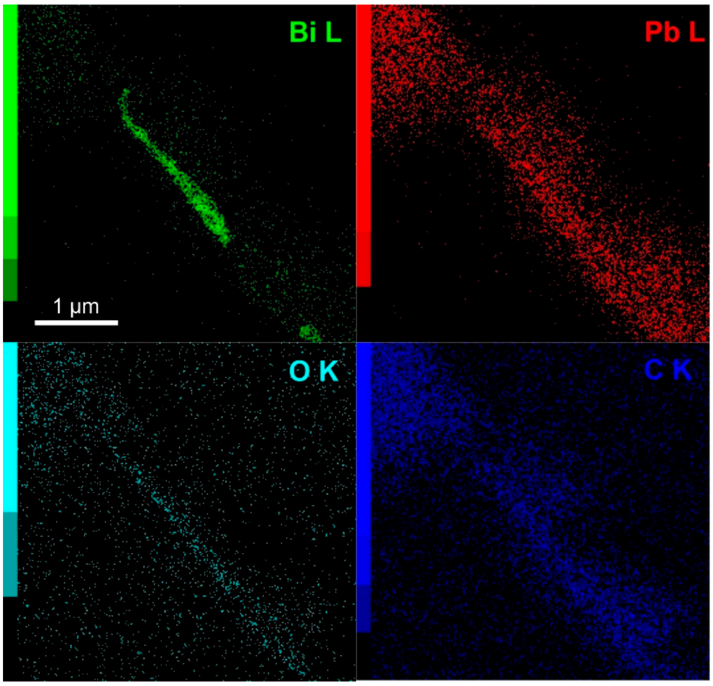

Figure S8. Energy dispersive X-ray spectroscopy maps of the contorted Pb- and Bi-holding whisker formed under high-oxygen environment.

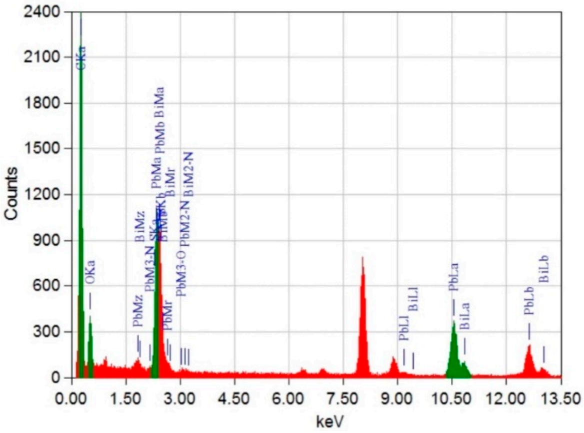

Figure S9. Example of energy dispersive X-ray spectroscopy spectra from a Pb- and Bi-holding whisker indicating the separability of the Pb and Bi signal from each other as well as the overall dominance of the Pb presence over Bi presence.

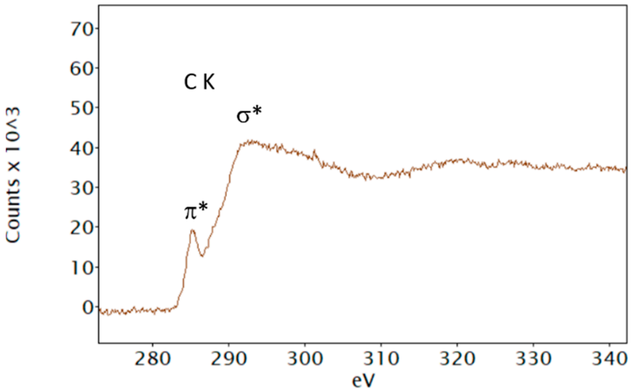

Figure S10. Electron energy loss spectroscopy (EELS) within the characteristic carbon range for individual whiskers with carbonous layer formed after 7-days exposure in high-oxygen and humidity environment.

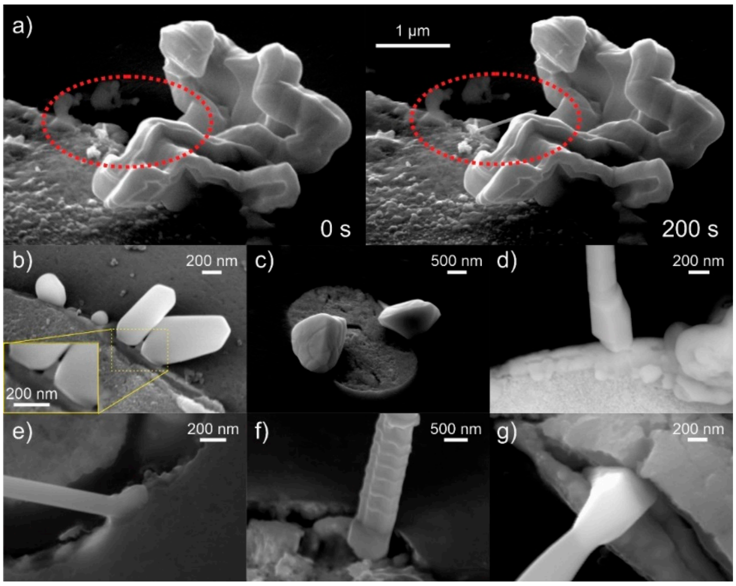

Figure S11. (**a**) Scanning electron microscopy (SEM) images depicting the instantaneous growth of a thin whisker with an approximate length of 800 nm in the time span of 200 s. (**b**) SEM image of plate-like whisker structures with dissimilar base. The enlargement indicates the unordered character of the base of the structure. (**c**) SEM image of a blocky spherical structure with segmentations. Note that the growth base of the structure is significantly smaller than the cross-section of the structure. (**d**) SEM image of a faceted whisker that displays varying faceting and orientation across its length. (**e**) SEM image of a smooth whisker with enlarged base. (**f**) Segmented whisker formation with striation that correspond to the individual growth spurts of the whisker during its growth process. (**g**) SEM image of a whisker with narrowing and broadening features through its length without temporally modified whisker base.

Supplementary video S1: Video of hillocks and initial whisker growth obtained with scanning electron microscopy. The acquisition time of each frame is 10 s and the duration of the video is 350 s.

Supplementary video S2: Video of sectional growth of faceted whisker and subsequent monotonic widening obtained with scanning electron microscopy. The acquisition time of each frame is 23 s and the duration of the video is 851 s.
